# To connect or not connect: long-term adoption of video consultations, and reasons for discontinuing use

**DOI:** 10.1177/1357633X231203267

**Published:** 2023-10-03

**Authors:** Irene Muli, Helena Hvitfeldt, Åsa Cajander, Lovisa Jäderlund Hagstedt, Nadia Davoody, Marina Taloyan, Maria Hägglund

**Affiliations:** 1 Participatory eHealth and Health Data Research Group, Department of Women's and Children's Health, 8097 Uppsala University, Uppsala, Sweden; 2 Department of Information Technology, 8097 Uppsala University, Uppsala, Sweden; 3 Health Informatics Centre, Department of Learning, Informatics, Management and Ethics, 27106 Karolinska Institutet, Stockholm, Sweden; 4 Academic Primary Healthcare Centre, Region Stockholm, Department of Neurobiology, Care Sciences and Society, 27106 Karolinska Institutet, Stockholm, Sweden; 5 59561 Biomedical Engineeering & Physics, Uppsala University Hospital, Uppsala, Sweden

**Keywords:** video consultations, eHealth, primary care, survey, Sweden, telehealth, willingness to practice‌

## Abstract

**Introduction:**

This study investigates factors related to long-term and short-term adoption of video consultations (VCs) and reasons for discontinuing use among primary care patients.

**Methods:**

A sample of primary care patients using VCs with healthcare providers were invited to take a survey in a cross-sectional study. Participants were asked about their intention to continue to have video consultations in the future, and those indicating no intention to use VCs in the future (short-term adopters) were asked about their reasons for this. Prevalence and statistical differences between long-term and short-term adopters were investigated.

**Results:**

There were several statistically significant differences between long-term and short-term adopters (76% vs. 24%). Long-term adopters consisted of more middle-aged individuals (35–54 years) and the majority worked full-time (56%). They had more positive opinions of VCs and used VCs and video meetings for other purposes to a larger extent. They chose VCs because of the lack of time to go to the healthcare centre and because their provider offered them. The most common reason for discontinuing use was a preference for face-to-face consultations, with the youngest age group (16–34 years) reporting this to a larger extent.

**Discussion:**

Younger and older age groups may be less likely to continue the use of VCs, potentially preserving the digital divide. Additionally, disparities in using similar technologies might contribute to the digital divide. Moreover, convenience, positive opinions of VCs, and experience with VCs were related to long-term adoption. Further studies are needed to explore non-use, age’s influence, and address usability issues.

## Introduction

Digital healthcare is becoming increasingly prevalent, and video consultations (VCs) are a significant component of this shift. For example, VCs use in healthcare is increasing, and the COVID-19 pandemic has further accelerated the implementation of these eHealth services.^1,2^ In Sweden, VCs were initially provided through online care applications in privately run healthcare, but publicly run healthcare has in the past few years developed competing online applications. The VC service *Alltid öppet* (“always open”) is an example of such an application, which was developed by the public healthcare provider in Region Stockholm, and was widely implemented at public primary care centres throughout the region during the pandemic. While over 90% of the Swedish population have access to and uses the internet, only 10% had received digital consultations (DCs), such as a VC with a physician in 2019,^3^ but use has since increased to 24% in 2022.^4^

Healthcare professionals and patients are highly satisfied with VCs.^5,6^ Benefits include improved accessibility for people with mobility or mental issues, reduced travel expenses, and reduced waiting times.^6^ Among Swedes, DCs such as VCs meant more accessible, effective care, and greater freedom.^7^ However, despite the reported benefits of VCs, some patients still prefer face-to-face consultations over VCs.^5,6^ In a study investigating the preferred amount of VCs in the future, patients preferred 49% of the care in the future to be provided via video.^8^

One of the early studies investigating patients’ willingness to accept an invitation to have a VC found that those willing to have a VC were slightly younger, were more comfortable setting up a VC, and had a longer distance to the face-to-face consultation than those not willing to have a VC.^9^ In general, those with experience with video calls were more comfortable setting up a VC. Another study among parents of children in oncology found that the best prediction of intention to use VCs in the future was, experience using other telecommunication technologies.^10^

Studies on the actual use of digital healthcare services (DHSs) such as VCs also raise concerns about the digital divide.^11^ Disparities in using VCs have been observed in relation to age and ethnicity.^12,13^ In Sweden, previous studies have shown younger people are more likely to use DCs, as are women, and households with children.^3,7,14^ People with higher socio-economic status, from metropolitan areas as well as people born in Sweden and have a history of chronic obstructive pulmonary disease/asthma or depression are also likely to be users.^14,15^

While numerous studies have examined the factors associated with VC use, no research has been found investigating the factors linked to the intention to continue using VCs after having used it at least once in primary care. Investigating factors associated with the intention to continue to use VCs might provide a piece to the puzzle of the causes of disparities in VC use and how these disparities might look in the future. Continued use, or long-term adoption, is also especially important in light of the COVID-19 pandemic's impact on VC use, as many patients have now overcome the initial barriers to using VCs, but we have little knowledge on whether they will continue to use VCs or not. Primary care context on the other hand is important since as the main point of healthcare contact for most patients, unequal access to its services has the potential to negatively influence public health.

### Aim

This study investigates factors related to long-term adoption (i.e., the intention to continue to have VCs) and short-term adoption (i.e., the intention to not have further VCs), as well as the reasons for discontinuing use among primary care patients who have had a VC during the COVID-19 pandemic.

## Methods

The study was given ethical approval by the Swedish Ethical Review Authority (reference number 2021-05096). Data for this cross-sectional study was collected through a survey. An invitation to participate in the study was sent through a text message to patients 16 years and older who had a VC through the *Alltid öppet* application from March to May 2022. In Sweden, young people can seek care and participate in research without parental consent from the age of 16 years old. Participants were sent two reminders where they were asked to disregard the reminders if they already had participated. A total of 4263 individual users that had VCs through *Alltid öppet* with healthcare professionals at 10 healthcare centres in Region Stockholm (5 centres representing different socio-economic areas in an urban municipality and 5 in a more rural municipality) were invited to participate. The healthcare centres were purposefully chosen to reach a diverse patient population. In the invite, participants were provided with a link to the study web page providing information about the study. Participants could download a file with complete study information. A link to the survey was provided at the bottom of the study web page. Consent to participate was explicitly assumed if participants proceeded to complete the survey. The survey was anonymous.

Swedish Primary care patients using *Alltid öppet* were chosen for this study because they represent a diverse demographic that has been at the forefront of healthcare digitisation, providing a holistic insight into the factors affecting both the uptake and discontinuation of VCs in a real-world setting.

The 40-question survey was developed by the research group consisting of experts in eHealth and primary healthcare and discussion with a reference group of other relevant experts and patient representatives. The survey was validated through two sets of cognitive interviews with people with experience of having VCs through *Alltid öppet*^16^ and pilot-tested once before the launch. Data was collected through the survey management platform Redcap (Research electronic data capture).^17^

The survey consisted of questions in Swedish regarding:

- The nature and experience of the latest VCs through ‘Alltid öppet’ – adapted from recommendations from The National Board of Health^18^
- Usability – adapted from the UMUX Lite items^19^
- Opinions of VCs and thoughts about future use
- Use of other online services – adapted from the annual Swedish survey “The Swedes and the Internet”^20^
- Health status and characteristics of the participants – adapted from the National Swedish Public Health Survey^21^

## Variables and analysis

### The independent variable

This study primarily focused on the participants’ intention to use VCs in the future, which was assessed by asking participants whether they would like to have VCs in the future with response options ‘yes’, ‘no’, or ‘don't know’. Participants responding ‘yes’, defined as long-term adopters, were compared to participants responding ‘no’ and ‘don’t know’, defined as short-term adopters.

### Explanatory variables

The long-term adopters and short-term adopters were compared regarding gender, age, education, working status, self-reported health, chronic illness, household constellation, place of birth, internet use, opinions of VCs, reasons for choosing VCs instead of a face-to-face consultation, use of VCs via *Alltid öppet* the past 12 months, use of VCs via other applications, and use of video meetings for other purposes (the items can be found in Appendix A). Responses ‘other/don’t know/don’t want to answer’ for different questions were excluded because they were too few. Other small responses were grouped, re-categorized, or sometimes removed. Related age groups were re-grouped to create three fairly proportional groups. All variables were categorical.

The rationale for use was measured by asking participants their reasons for choosing a VC instead of a face-to-face consultation with five predefined response options and one free-text option. The rationale for discontinuing use was measured using a follow-up question to participants who did not want to have more VCs in the future with seven predefined response options and one free-text option (the items can be found in Appendix B). Free-text answers were not analysed. Age distribution in the three most common reasons for discontinuing use was also analysed.

### Statistical analysis

This study had a descriptive and comparative approach. To test for the significance of the differences between long-term adopters and short-term adopters, chi-square tests or Fisher's exact tests were used. A p-value < 0.05 was considered significant. Post hoc analyses of significant variables were conducted using adjusted residuals and comparing cells with Bonferroni adjusted p-value. Due to the size of the groups, further analysis was deemed inappropriate. The statistical programme Stata 17 was used to conduct the analysis.

## Results

In total 4263 patients were invited to participate in the survey, where 528 participated, equalling a 12% response rate. Most of the participants (342/451, 76%) stated that they would like to have VCs in the future, while 17% (79/451) did not know, and 7% (30/451) did not want to. Hence 76% (342/451) of the participants represented the long-term adopters, and 24% (109/451) the short-term adopters.

**Figure 1.**
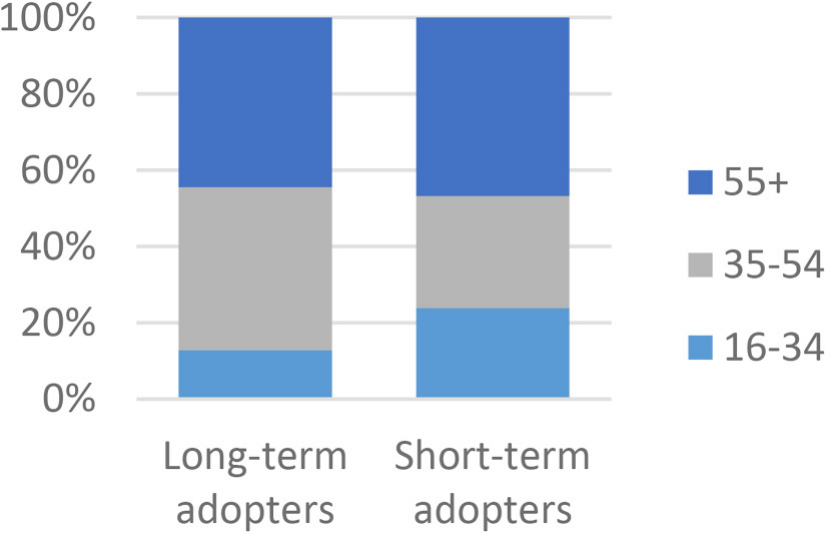
A comparison of long-term (n = 342) and short-term (n = 109) adopters in regards to Age, p = 0.006* N = 451.

**Figure 2.**
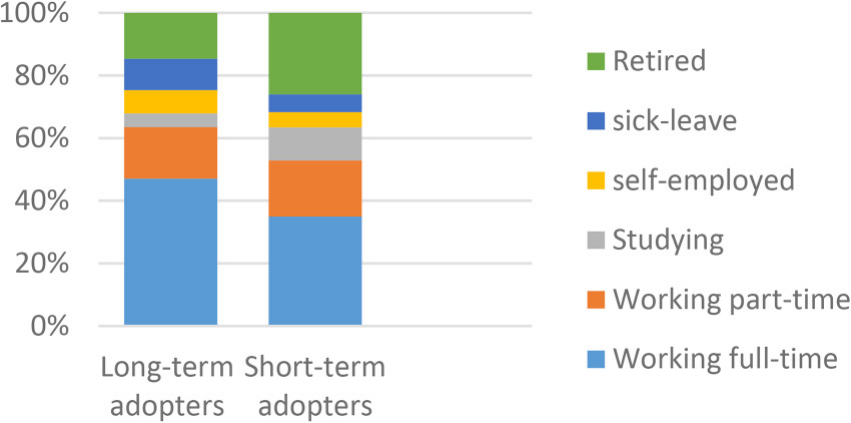
A comparison of long-term (n = 342) and short-term (n = 109) adopters in regards to working status, p = 0.016* N = 451.

There were statistically significant differences between the two groups in age and working status (Figures 1 and 2). Short-term adopters had a larger proportion of participants younger than 35 years (26/109, 24%) compared to long-term adopters (44/342, 13%) and a slightly larger proportion of participants 55  +  years (51/109, 46%). The majority of the long-term adopters had full-time work (183/342, 56%) while the biggest group of short-term adopters were retired (32/109, 39%). Post hoc analysis accredited the association for age mainly to age groups younger than 55 years. Association for working status was mainly driven by full-time work. Although most participants were female there were no significant differences related to gender.

**Figure 3.**
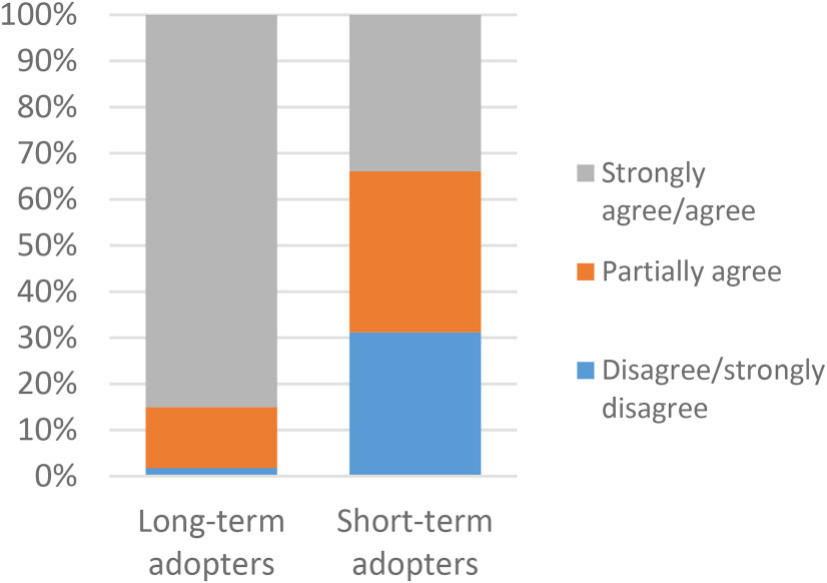
A comparison of long-term (n = 341) and short-term (n = 109) adopters in regards to video consultations are a good development. p = 0.000* N = 450.

**Figure 4.**
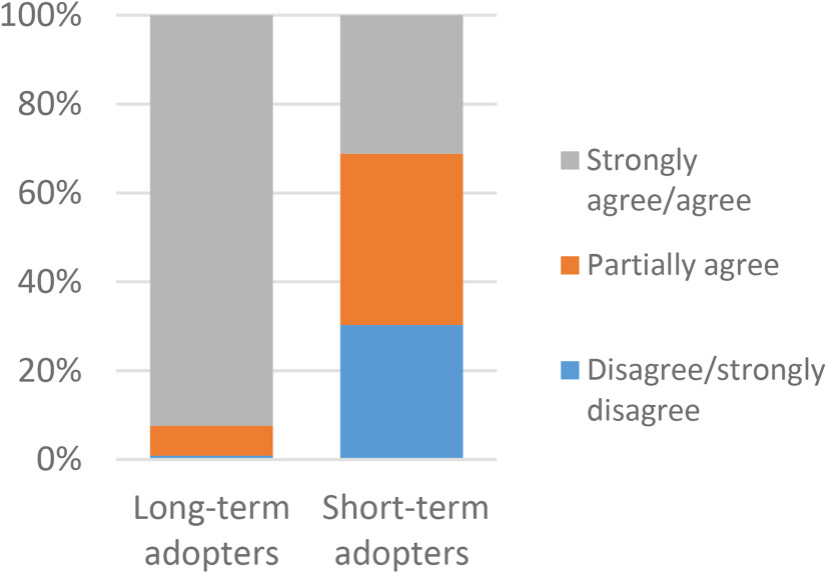
A comparison of long-term (n = 342) and short-term (n = 109) adopters in regards to It is good for me to have the opportunity, p = 0.000* N = 451.

When asked if VCs are a good development, most long-term adopters agreed or strongly agreed (290/341, 85%), while most short-term adopters partially agreed (38/109, 35%) or disagreed (34/109, 31%) (Figure 3). When asked if it was a good opportunity for them to have VCs in healthcare almost all long-term adopters agreed or strongly agreed (316/342, 92%), while most short-term adopters partially agreed (42/109, 39%) or disagreed (33/109, 30%) (Figure 4). According to the post hoc analysis, all the responses significantly contribute to the associations.

**Figure 5.**
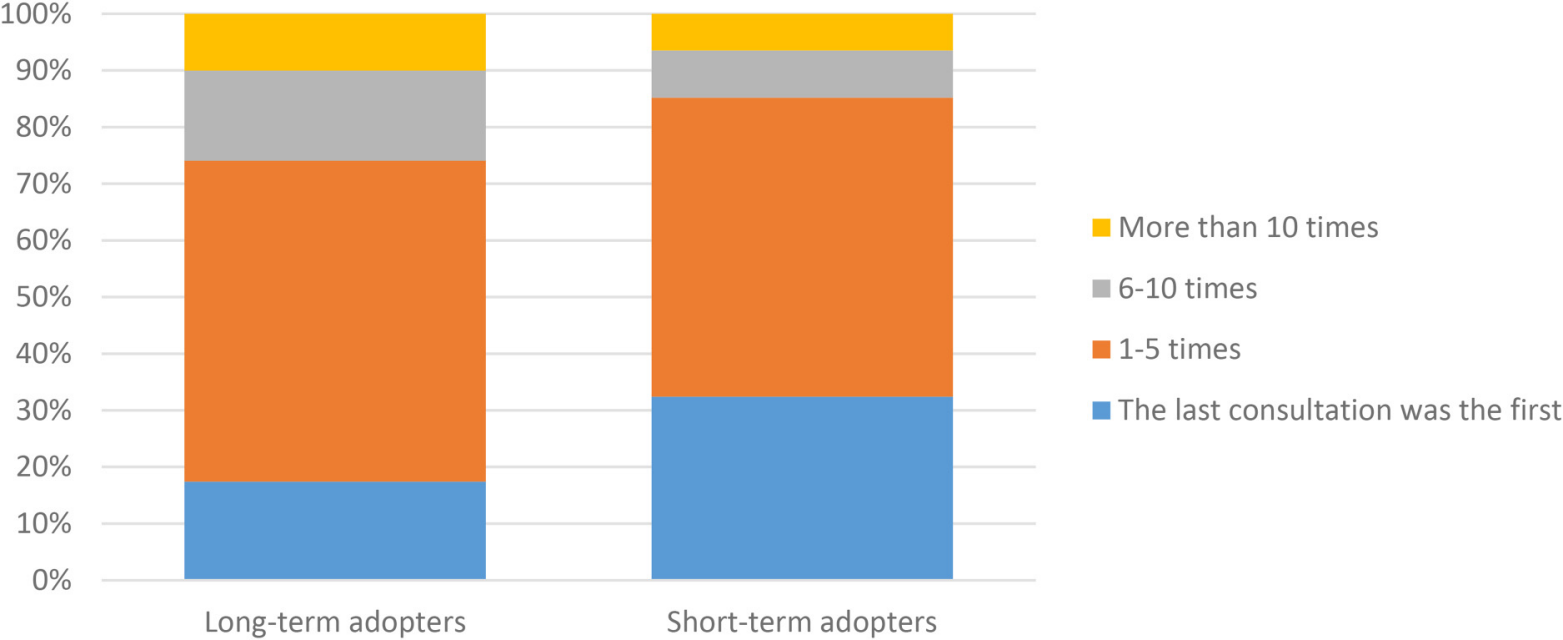
A comparison of long-term (n = 339) and short-term (n = 108) adopters in regards to use of video consultations through ‘Alltid Öppet’ for the last 12 months, p = 0.004* N = 447.

**Figure 6.**
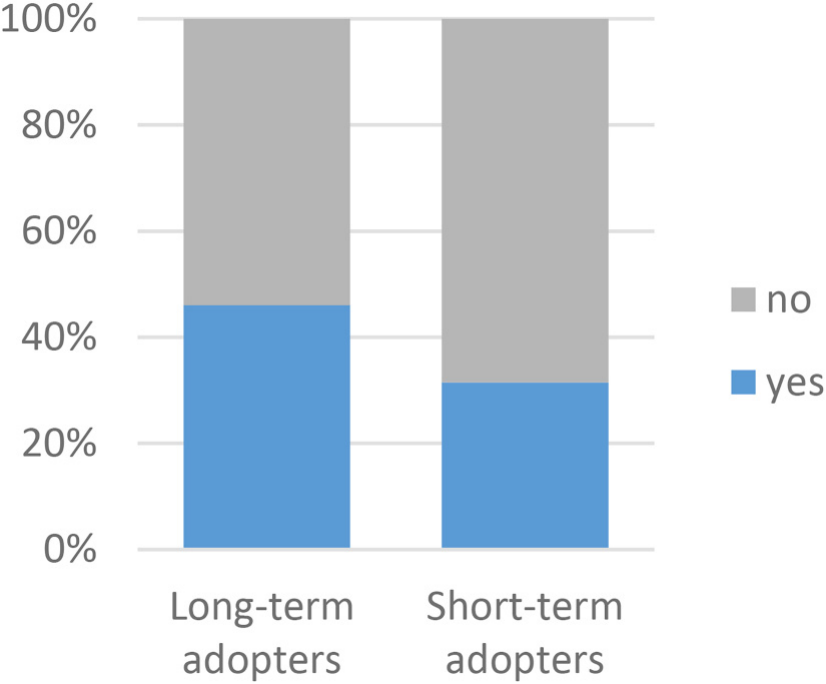
A comparison of long-term (n = 341) and short-term (n = 108) adopters in regards to use other applications for VCs, p = 0.008* N = 449.

**Figure 7.**
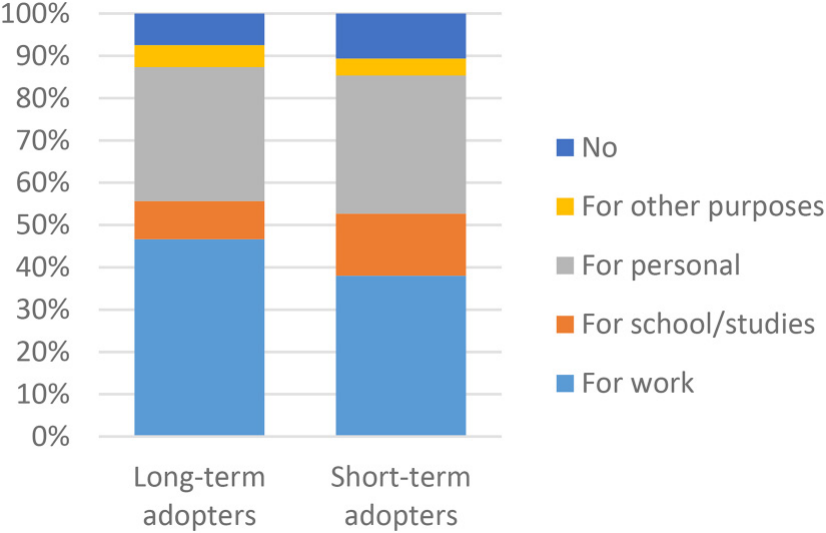
A comparison of long-term (n = 342) and short-term (n = 109) adopters in regards to use of video meetings for other purposes, p = 0.021* N = 451.

There were also significant differences between the groups regarding the use of VCs via ‘*Alltid öppet’* in the past 12 months (Figure 5). Long-term adopters were more frequent users with 26% of them having more than 5 VCs versus 16% among short-term adopters. Post hoc analysis accredited the association mainly to new users. They also had more VCs via other applications (46% vs 31%) and used video meetings for work (71%) and for personal reasons (49%) (Figures 6 and 7). Short-term adopters on the other hand used video meetings for school/studies (20%) and did not use them at all (15%) to a greater extent than long-term adopters. Post hoc analysis accredited the association mainly to the use of video meetings for work (see Appendix C for a complete table of the comparisons).

**Figure 8.**
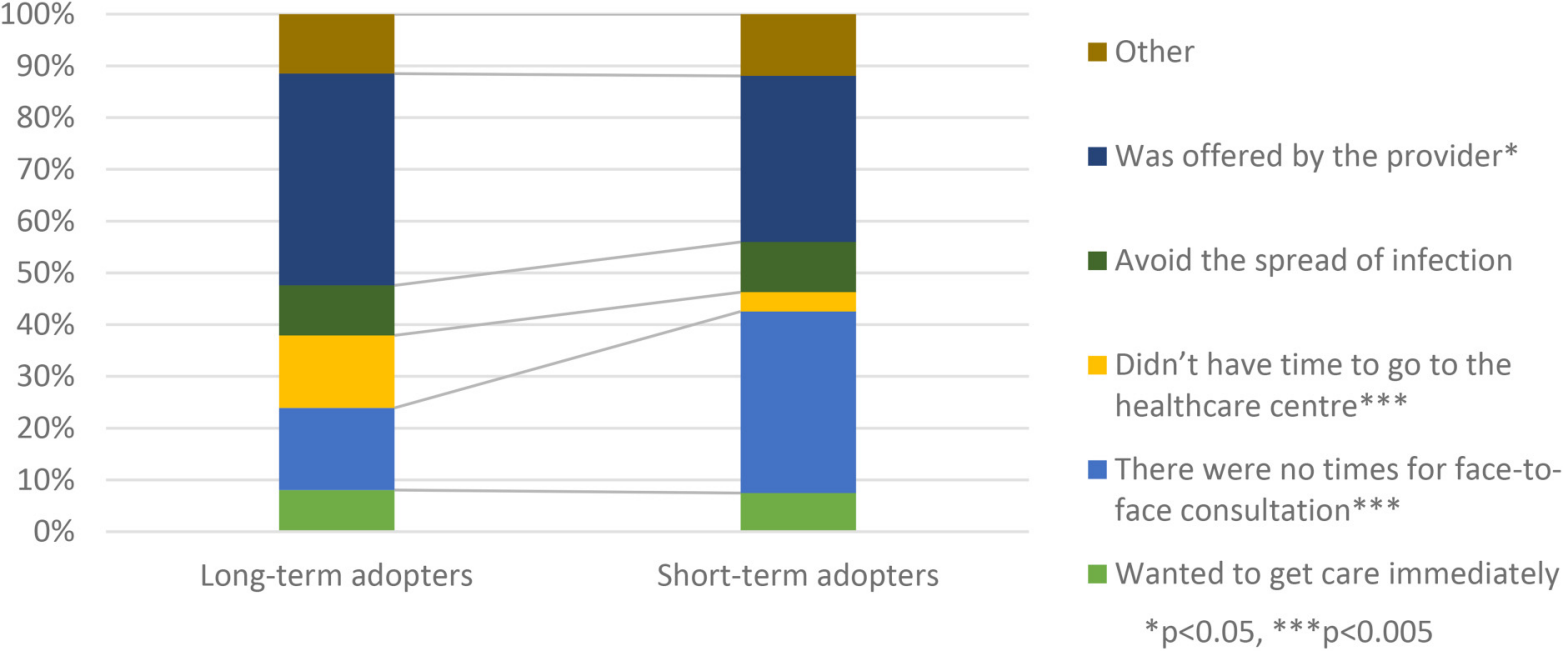
A comparison of long-term (n = 342) and short-term (n = 109) adopters in regards to reason for choosing video consultation N = 451.

Three significant differences were found regarding reasons for choosing VC instead of a face-to-face consultation (Figure 8). Long-term adopters chose VC because of the lack of time to go to the centre and because they were offered by their provider to a larger extent than short-term adopters, while short-term adopters reported the non-availability of face-to-face consultations as the reason to a larger extent than long-term adopters.

**Figure 9.**
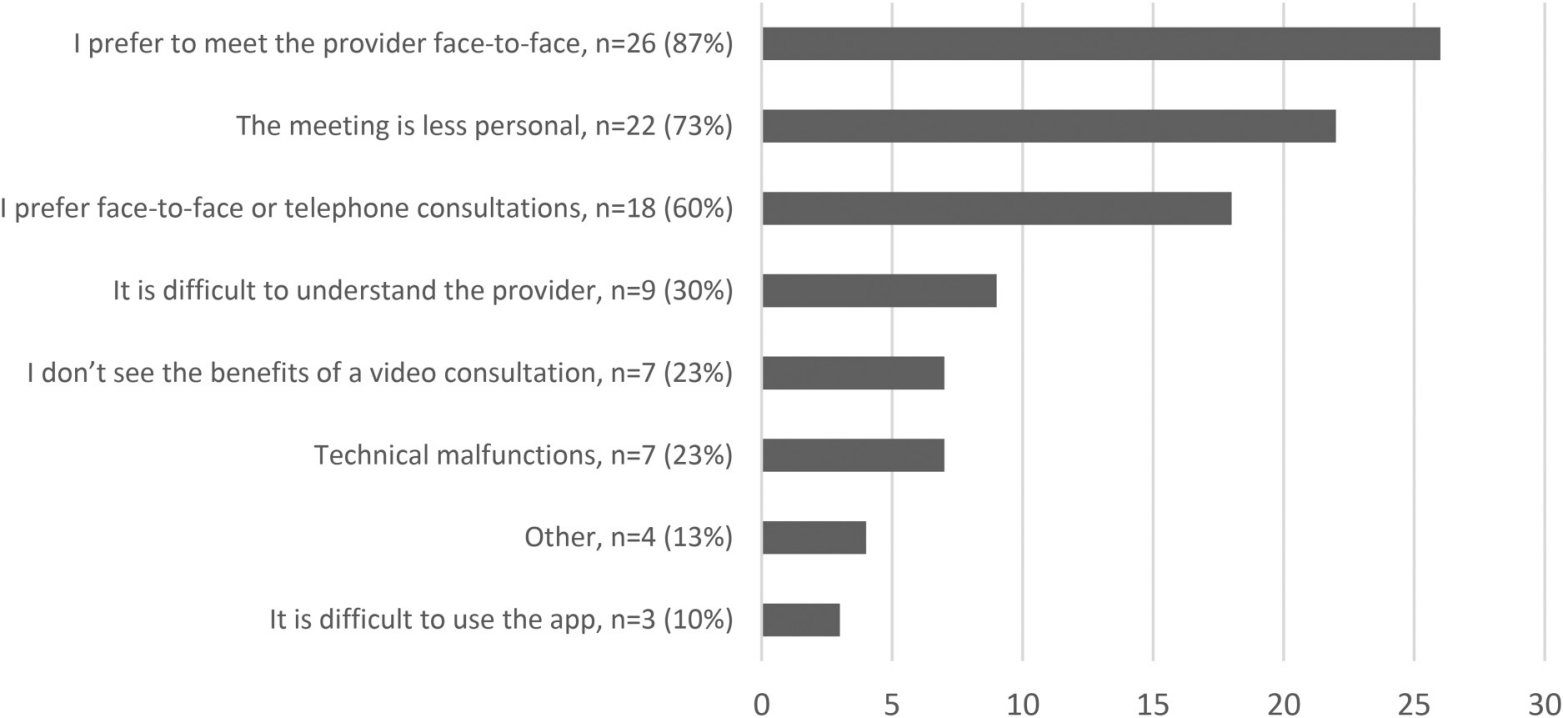
Reasons for not wanting more video consultation N = 30.

The most common reason for not wanting VCs in the future was a preference to meet the healthcare professionals face-to-face (26/30, 87%), followed by VCs being less personal (22/30, 73%), and preferring a face-to-face or telephone consultation (18/30, 60%) (Figure 9). Reasons related to usability were less common.

**Figure 10.**
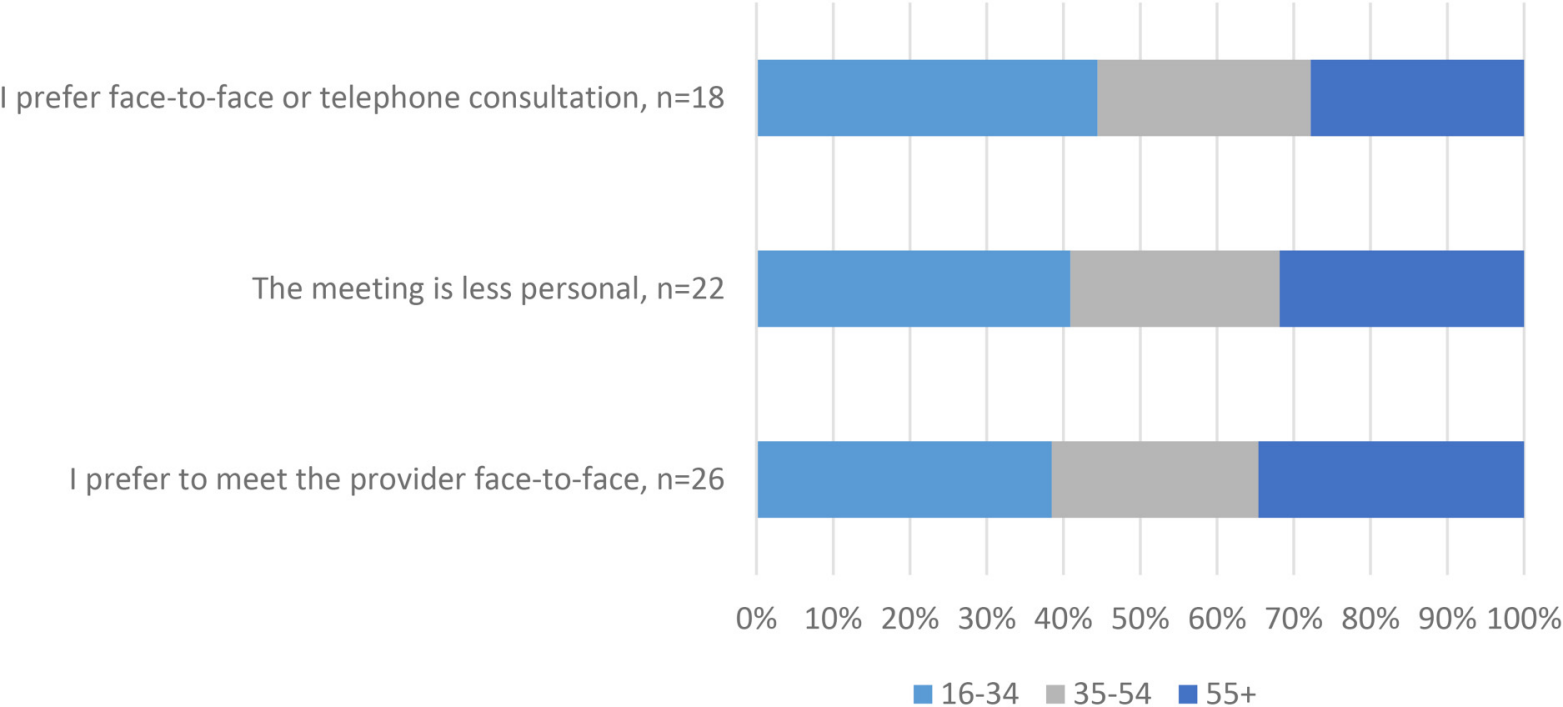
The three most common reasons for not wanting more VCs by age group.

The largest age group among the three most common reasons for not wanting more VCs was 16- to 34-year-olds followed by 55 + -year-olds (Figure 10).

## Discussion

This study aimed to investigate factors related to long-term and short-term adoption of VCs and explore the rationale for discontinuing use among primary care patients. There were significant differences between the groups in socio-demographics (age and working status), opinions of VCs, use of VCs via the *Alltid öppet* and other applications, and the reason for choosing VC. The most common reason for not wanting to have more VCs was because they preferred to meet the healthcare professionals face-to-face, with the youngest age group (16–34 years) reporting this to a larger extent.

### Factors related to long-term/short-term adoption

Short-term adopters consisted of significantly more individuals younger than 35 years and older than 54 years compared to long-term adopters. These results could indicate a decreased interest in and/or use of VCs among these groups in the future, potentially increasing the digital divide. Similar results have been reported in one study looking at telehealth users in general,^12^ but in most studies, non-users are generally older.^13,14^ This association of non-use and old age could be explained by the fact that some studies use only two age groups,^13^ and others include children as users when parents are the actual users.^7,14^ Moreover, according to The Unified Theory of Acceptance and Use of Technology (UTAUT), age is one of the mediating factors affecting the use and intention to use technology^22^; younger age amplifies the effects of performance expectancy on intention to use while older age amplifies social influence effects on intention to use technology. Further studies are needed to explore age's influence on the intention to continue to have VCs.

Convenience also seems to be an important factor for long-term adoption, people working full-time, self-employed and people on sick leave might not be able to go to a face-to-face consultation to the same extent as those who work part-time, study, or are retired. Having full-time work has also previously been shown to be associated with use.^23^ Having to suffer economic loss or having other commitments have similarly been linked to willingness to have VCs.^9^

Significantly more long-term adopters than short-term adopters thought VCs in healthcare were a good development and a good opportunity for them. These results could reflect general opinions related to the perceived usefulness of VCs or opinions based on negative user experiences. In one study, elderly Swedish non-users reported many opinions related to barriers to use, such as scepticism towards VCs and how they might be used to exploit the reimbursement system, having no need for digital care, and thinking that it was for the younger generation.^24^ Usability issues have also been reported where in some cases issues with VCs led to a switch to telephone consultation^5,25^ potentially affecting users’ opinions of VCs. A deeper exploration of the opinions of VCs and their impact on long-term adoption is needed.

The long-term adoption group had more frequent users of VCs, while short-term adopters were more often new users, which could reflect the previously mentioned usability issues. Long-term adopters also reported having VCs via other applications to a higher degree than short-term adopters, indicating more experience and potentially reflecting a more positive opinion of VCs in general. Similar associations were observed in one study where comfort in setting up VCs was the main factor associated with willingness to have VCs.^9^ Experience with VC has also previously been associated with choosing VC^26^ and since comfort with having VC comes with experience an association between frequent use and long-term adoption is not surprising. According to UTAUT experience is also a factor that moderates the intention to use technology,^22^ limited experience amplifies the effects of effort expectancy and social influence on the intention to use.

Long-term adopters were also more likely to use video meetings for work and slightly more likely to use them for personal and other purposes. Short-term adopters on the other hand were more likely to use video meetings for school/studies or not at all. Similar associations were observed in a previous study^10^ where using similar technology was associated with the intention to use.

The identified differences in opinions of VCs and previous experience with similar technologies among long-term and short-term adopters may indicate that existing disparities in use will not disappear on their own. Interventions aiming to reduce the digital divide may want to consider these factors.

### Reasons for use and discontinuing use

Long-term adopters were more likely to choose VCs because they didn’t have time to go to the healthcare centre or because the provider offered them a VC compared to short-term adopters. Short-term adopters had on the other hand a higher proportion of participants who chose VCs because face-to-face consultations were unavailable. Convenience has previously been stated as one of the benefits of DCs and VCs^23^ and it is thus not surprising that choosing a VC to save time was associated with continued intention to use VCs. This study also suggests that the issues with waiting times in Sweden^27^ are driving the adoption of VCs among patients, as patients choose VCs rather than wait for a face-to-face consultation. Furthermore, given that the experience of having VCs is associated with the likelihood of use in the future, this could lead to increased adoption of VCs.

In contrast, feeling forced to use VCs due to a lack of face-to-face appointments may negatively impact patients’ opinions of VCs. According to UTAUT voluntariness is also a factor that influences intention to use,^22^ and mandatory use amplifies the effects of social influence on intention to use technology. If patients feel like their only option is to use VCs, this likely affects their feeling of voluntariness.

The main reason for not wanting VCs in the future was that participants preferred to meet the healthcare professionals face-to-face. This reason was most common among younger respondents (16–34 years). These results are not surprising, VC users have also previously reported preferring face-to-face meetings.^28,29^ This could be related to continuity and reasons for contact, factors that have previously been associated with the perceived suitability of VCs.^30^ In other words, VCs were not deemed suitable for all cases handled in primary care. Studies are needed to further explore long-term adoption in relation to new contacts versus follow-ups and mental versus somatic reasons for contact. Remarkably, usability issues were not among the main barriers for future use reported by our respondents, but although small they should be explored further and addressed. The fact that the largest age group among responders of the most common reasons for discontinuing use is 16 to 34 years is also unforeseen and should be explored in depth.

## Strengths and limitations

As a cross-sectional study, there is always a risk for season-related bias. Although participants were recruited from healthcare centres in both a rural and an urban municipality, there is still a risk for selection bias and a risk that the sample might not be a good representation of the users of VCs in general. Recruiting participants from one publicly run application only might also have skewed the results. Since the survey was in Swedish, users who are not native speakers might also be deterred from participating. A strength on the other hand is that this study included both first-time users and those who have been using VCs for a while, making it possible to assess long-term adoption for users with varying experiences. Another strength of this study is that the survey was validated with VC users and a reference group, but a limitation is that the validation sample was small and validation rounds were few.

The anonymity of the survey is a strength. Still, it also meant a risk for duplication of the responses where the participant, when reminded to participate, could fill out the survey again. Yet, the risk is perceived to be minimal. Since the survey was anonymous, the non-responders could also not be analysed. The low response rate and the subsequent sample size is also a limitation of this study. Since the study sample size was limited, advanced analysis of the results could not be conducted.

While investigating the intention to continue using VCs, there can be a discrepancy between intention and behaviour. In addition, factors such as cost, availability, and geographical location may affect long-term adoption, which were not investigated in this study. Further studies are therefore needed to investigate the association between intention to use and the actual use of VCs and the factors that affect it. The question about the intention itself might also have been difficult to respond to as one might be willing to have VC in the future in some cases but not others.

## Conclusion

This study suggests that younger and older age groups may be less likely to use VCs long-term, potentially preserving the digital divide. There is also an indication that disparities in the use of similar technologies might contribute to the digital divide. Convenience, opinions of VCs, and experience with VCs on the other hand were related to long-term adoption. At the same time, preference for face-to-face consultations was the main barrier to VC use, especially among those aged 16 to 34. Further studies are needed to explore non-use, age's influence, and address usability issues. Overall, this study provides valuable information for improving the adoption of VCs in primary care.

## Appendix

**Appendix A.** The explanatory variables based on survey questions and response alternatives

Variables	Questions	Options	Analysed categories
Gender	Gender	Female Male Other	Female Male
Age	How old are you?	16–18 19–24 25–34 35–44 45–54 55–64 65–74 75–84 85 or older	16–34 35–54 55 +
Working status	What is your main occupation?	Working full-time Working part-time Studying Self-employed Sick leave Retired Parental-leave Don’t know/don’t want to answer	Working full-time Working part-time Studying Self-employed Sick leave Retired
Education	What is your highest completed education?	Primary education or similar High school 2 years High school 3–4 years Adult school University or college <2 years University or college 3 years or longer Don’t know/don’t want to answer	< = 12 > = 13
Household	Who do you live with? In other words that you live with at least half the time	No one Parents or siblings Partner Other adults Children under 18 years	No one Parents or siblings Partner Other adults Children under 18 years
Health	How do you assess your general health?	Good Very good Neither good nor bad Bad Very bad	Good or very good Neither good nor bad Bad or very bad
Chronic illness	Do you have a chronic illness or any other chronic health problem	No Yes	No Yes
Place of birth	Where are you born?	Sweden Other Nordic country Other European country Outside Europe Don’t want to say	Sweden Other countries
Internet use	How often do you use the internet with the following devices? (mobile phone, computer, tablet)	Daily Every week Every month Sometimes Never	Daily Less or Never
Good development	Do you think the implementation of video consultations is a good development?	Strongly agree Agree Partially agree Disagree Strongly disagree	Strongly agree/agree Partially agree Disagree/strongly disagree
Good opportunity	It is good for me to have the opportunity to have video consultations with healthcare professionals	Strongly agree Agree Partially agree Disagree Strongly disagree	Strongly agree/agree Partially agree Disagree/strongly disagree
Use the past 12 months	How many times have you had a video consultation through *Alltid öppet* the past 12 months	Last consultation was the first 1–5 times 6–10 times More than 10 times	Last consultation was the first 1–5 times 6–10 times More than 10 times
Use other applications for video consultations	Have you had VCs through other applications other than Alltid öppet	Yes, 1–5 times Yes, 6–10 times Yes more than 10 times No	Yes No
Have video for other purposes	Do you have video meetings for other purposes(e.g., zoom, teams, skype, FaceTime)	For work For school/studies For personal For other purpose[free text] No	For work For school/studies For personal For other purposes No

## Appendix

**Appendix B.** Reasons for useing and discountinuing use of video consultations measurements

	Question	Options
Reason for use	Why did you choose a video consultation instead of a face-to-face consultation?	Wanted to get healthcare immediately There were no available times for face-to-face consultation Didn’t have time to go to the healthcare centre Avoid the spread of infection Was offered by the provider Other [free text]
Reason for non-use	What are the main reasons for not wanting to have more video consultations?	Technical malfunctions I prefer face-to-face or telephone consultations It is more difficult to understand the provider I prefer to meet the provider face-to-face The meeting is less personal I don’t see the benefits of a video consultation It is difficult to use the app Other [free text]

## Appendix

**Appendix C.** Prevalence and a comparison of long-term and short-term adopters, n  =  451

	Total	Long-term adopters	Short-term adopters	p
	n (%)	n (%)	n (%)	
Gender				
Male	128 (29%)	96 (29%)	35 (33%)	0.691
Female	310 (71%)	238 (71%)	72 (67%)	
Age				
16–34	70 (16%)	44 (13%)	26 (24%)	0.006*
35–54	178 (39%)	146 (43%)	32 (29%)	
55 +	203 (45%)	152 (44%)	51 (46%)	
Education				
< = 12	163 (37%)	120 (36%)	43 (41%)	0.389
> = 13	277 (63%)	214 (64%)	63 (59%)	
Working status				
Working full-time	226 (52%)	183 (56%)	43 (41%)	0.016*
Working part-time	86 (20%)	64 (19%)	22 (21%)	
Studying	30 (7%)	17 (5%)	13 (12%)	
Self-employed	35 (8%)	29 (9%)	6 (6%)	
Sick leave	46 (11%)	39 (12%)	7 (7%)	
Retired	89 (21%)	57 (17%)	32 (39%)	
Health				
Good or very good	253 (57%)	193 (57%)	60 (56%)	0.962
Neither good nor bad	129 (29%)	98 (29%)	31 (29%)	
Bad or very bad	63 (14%)	47 (14%)	16 (15%)	
Chronic illness				
No	175 (39%)	125 (37%)	50 (47%)	0.072
Yes	270 (61%)	213 (63%)	57 (53%)	
Place of birth				
Sweden	399 (90%)	307 (91%)	92 (88%)	0.5073
Other countries	43 (10%)	31 (9%)	12 (12%)	
Household constellation				
No one else	96 (21%)	74 (22%)	22 (21%)	0.058
Parent or sibling	18 (4%)	8 (2%)	10 (9%)	
Spouse	290 (65%)	222 (67%)	68 (64%)	
Other adults	41 (9%)	34 (10%)	7 (7%)	
Children under 18 years	114 (26%)	93 (28%)	21 (20%)	
Use of the internet on mobile phone				
Daily	452 (98%)	334 (98%)	103 (96%)	0.229
Less or never	10 (2%)	6 (2%)	4 (4%)	
Use of the internet on a computer				
Daily	275 (65%)	213 (66%)	62 (62%)	0.470
Less or never	148 (35%)	110 (34%)	38 (38%)	
Use of the internet on a tablet				
Daily	121 (30%)	95 (30%)	26 (27%)	0.503
Less or never	289(70%)	218 (70%)	71 (73%)	
Video consultations are a good development				
Disagree/strongly disagree	40 (9%)	6 (2%)	34 (31%)	0.000*
Partially agree	83 (18%)	45 (13%)	38 (35%	
Strongly agree/agree	327 (73%)	290 (85%)	37 (34%)	
It is good for me to have the opportunity				
Disagree/strongly disagree	350 (78%)	3 (1%)	33 (30%)	0.000*
Partially agree	65 (14%)	23 (7%)	42 (39%)	
Strongly agree/agree	36 (8%)	316 (92%)	34 (31%)	
Video consultations through “*Alltid öppet*” for the past 12 months				0.004*
The last consultation was the first	94 (21%)	59 (17%)	35 (32%)	
1–5 times	251(56%)	192(57%)	57 (53%)	
6–10 times	63 (14%)	54 (16%)	9 (8%)	
More than 10 times	41 (9%)	34 (10%)	7 (6%)	
Use other applications for video consultations				0.008*
Yes	191 (43%)	157 (46%)	34(31%)	
No	258 (57%)	184 (54%)	74(69%)	
Have video meetings for other purposes				
For work	301 (67%)	243 (71%)	57 (53%)	0.021*
For school/studies	69 (15%)	47 (14%)	22 (20%)	
For personal	215 (48%)	165 (49%)	49 (45%)	
For other purposes	33 (7%)	27 (8%)	6 (6%)	
No	55 (12%)	39 (11%)	16 (15%)	

*Statically significant

Chi-square tests were conducted to test for the significance of the differences. A p-value < 0.05 was considered significant. Working status and household constellation were multiple-choice answers.
